# A rare case of undifferentiated embryonal sarcoma of the liver in an elderly patient misdiagnosed as intrahepatic cholangiocarcinoma

**DOI:** 10.1016/j.ijscr.2025.112059

**Published:** 2025-10-13

**Authors:** Xiang-Hui Geng, Deng-Shuai Li, Xing-Fu Wang, Wei Zhong, Min-Feng Liang, Jie Lin

**Affiliations:** a Department of General Surgery I, The People's Hospital of Jiashi County, Kashgar, 844300, Xinjiang, China; b Intensive Care Unit, The People's Hospital of Jiashi County, Kashgar, 844300, Xinjiang, China; c Department of Gastroenterology, The People's Hospital of Jiashi County, Kashgar, 844300, Xinjiang, China; d Administrative Office, The People's Hospital of Jiashi County, Kashgar, 844300, Xinjiang, China; e Department of Infectious Disease, The First People's Hospital of Foshan, Foshan, 528000, Guangdong, China; f Department of Hepatobiliary Pancreatic and Splenic Surgery, The Eighth Affiliated Hospital of Southern Medical University (The First People's Hospital of Shunde Foshan), Foshan, 528300, Guangdong, China

**Keywords:** Undifferentiated embryonal sarcoma of the liver (UESL), Intrahepatic cholangiocarcinoma, Case report, Elderly, Misdiagnosis, Liver neoplasm

## Abstract

**Introduction:**

Undifferentiated embryonal sarcoma of the liver (UESL) is an exceptionally rare and aggressive malignancy, predominantly affecting children. Its occurrence in the elderly is exceedingly uncommon, posing significant diagnostic challenges. We present the oldest-reported case of UESL, initially misdiagnosed as intrahepatic cholangiocarcinoma (iCCA).

**Presentation of case:**

An 88-year-old male presented with an upper abdominal mass and discomfort. Imaging (CT/MRI/PET-CT) and mildly elevated CA19–9 initially suggested iCCA (T1bN0M0, Stage IB). After 10 months of disease progression despite chemotherapy (capecitabine), left hemihepatectomy was performed. Histopathological examination revealed UESL (7.5 cm tumor; IHC: Vimentin+, CD10+, CD56+, Ki-67 80 %). The patient developed recurrent hepatic disease and pulmonary metastases 1.5 months postoperatively and succumbed to the disease shortly thereafter, with an overall survival of approximately one year.

**Discussion:**

UESL in octogenarians is exceptionally rare, with nonspecific imaging and serological findings (e.g., mild CA19–9 elevation) leading to frequent misdiagnosis, often as iCCA. Pathological examination remains essential for definitive diagnosis. Despite radical resection, prognosis in elderly patients is poor due to aggressive biology and limited tolerance for adjuvant therapy. This case underscores the importance of considering UESL in atypical liver masses across all age groups.

**Conclusion:**

UESL should be included in the differential diagnosis of atypical liver masses, even in elderly patients. Early surgical resection offers the best chance for survival, though outcomes remain dismal in advanced age due to rapid recurrence and metastasis. Heightened awareness of this entity is crucial to mitigate diagnostic delays.

## Highlights

• Oldest UESL case in an 88-year-old, expanding the age spectrum of this rare cancer.
• Misdiagnosed as iCCA due to atypical imaging & mild CA19-9 rise, showing UESL's diagnostic complexity in elderly.
• Rapid recurrence (liver & lung mets) 1.5 months post-surgery, poor prognosis in elderly despite resection.
• UESL confirmed by IHC (Vimentin, CD56+, Ki-67 80%) after inconclusive imaging/serology.
• Consider UESL in differentials for atypical liver masses in all ages to reduce diagnostic delay.

## 1 Introduction

Undifferentiated embryonal sarcoma of the liver (UESL) is a rare malignant tumor originating from undifferentiated mesenchymal cells, first described by Stocker and Ishak in 1978 [1]. Predominantly affecting children aged 6–10 years, UESL is exceptionally rare in adults, accounting for less than 0.2 % of primary liver malignancies [2]. The tumor's nonspecific clinical presentation, including abdominal pain, mass, or fever, combined with the lack of characteristic laboratory markers (e.g., normal AFP, occasional mild elevations in CA19–9 or CA125) and imaging findings (e.g., cystic-solid masses with necrosis on CT/MRI), contribute to a high preoperative misdiagnosis rate [[3], [4], [5]]. Differential diagnoses often include intrahepatic cholangiocarcinoma (iCCA), hepatic abscess, echinococcosis, hepatocellular carcinoma, or other sarcomas, making diagnosis challenging, especially in the elderly where comorbidities and atypical presentations exacerbate delays [[6], [7], [8]]. Treatment typically involves radical surgical resection as the cornerstone, with adjuvant chemotherapy improving survival in select cases, though liver transplantation may be considered for unresectable tumors [4,9,10]. The tumor's high malignancy and poor prognosis are well-documented [3]. In elderly patients, UESL is even rarer, with only a few documented cases, the oldest previously reported being an 86-year-old patient [11]. This report presents a case of an 88-year-old male with UESL misdiagnosed as intrahepatic cholangiocarcinoma, highlighting diagnostic challenges and the aggressive nature of the disease in the elderly. The work has been reported in line with the SCARE 2025 criteria [12].

## 2 Presentation of case

**Fig. 1:**
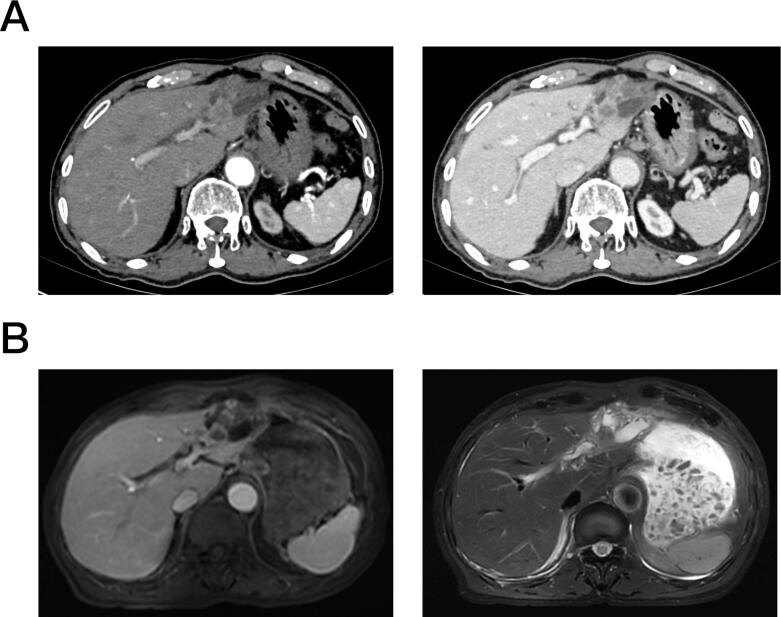
Preoperative abdominal contrast-enhanced CT (A, left: arterial phase, right: venous phase) and MRI (B, left: T1WI, right: T2WI) (April 2024) showing a 1.2 cm × 1.2 cm mass in the left lateral segment of the liver with heterogeneous enhancement and intrahepatic bile duct dilation.

**Fig. 2:**
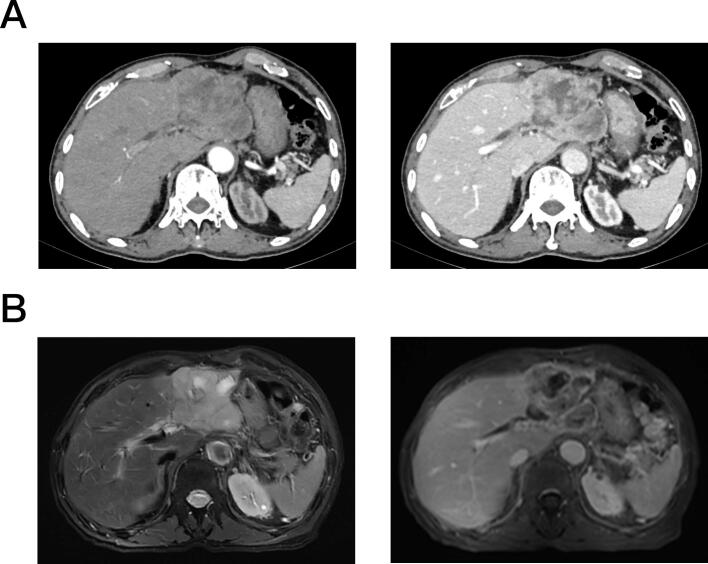
Preoperative abdominal contrast-enhanced CT (A, left: arterial phase, right: venous phase) and MRI (B, left: T1WI, right: T2WI) (February 2025) showing mass enlargement (7.3 cm × 5.6 cm).

An 88-year-old male was admitted to our hospital on February 20, 2025, with a 10-month history of an upper abdominal mass and 2 months of upper abdominal discomfort. He had a history of pulmonary tuberculosis but no other significant medical conditions. Ten months prior, an abdominal ultrasound revealed a low-echo mass in the left lateral segment of the liver (1.0 cm × 1.2 cm) with distal bile duct dilation. Tumor markers were normal at that time. Subsequent contrast-enhanced CT and MRI suggested intrahepatic cholangiocarcinoma (Fig. 1). PET-CT indicated possible cholangiocarcinoma with lymph node metastasis. The initial lesion was deemed resectable based on imaging; however, upfront surgery was deferred due to the patient's advanced age, frailty, history of pulmonary tuberculosis, and family preference for less invasive options. No pre-chemotherapy tissue diagnosis, such as ERCP-guided brush cytology or fluorescence in situ hybridization (FISH), was attempted, as the imaging features strongly supported iCCA, and invasive procedures were considered high-risk in this elderly patient. The patient received six cycles of chemotherapy with Capecitabine (Xeloda) 300 mg bid, as a bridge to potential surgery; however, follow-up imaging showed slight enlargement of the liver mass. Two months before admission, his symptoms worsened. Repeat imaging demonstrated significant growth of the liver mass (73 mm × 56 mm). Physical examination revealed no jaundice, and the abdomen was soft with no palpable masses. Laboratory tests showed a white blood cell count of 10.24 × 10^9^/L, neutrophil percentage of 68 %, CA19–9 of 52.61 U/mL, and normal levels of AFP, CA125, and CEA. Hepatitis B markers were negative, and liver and renal functions were normal. Chest CT showed multiple fibrotic strands and nodules in both lungs, consistent with old pulmonary tuberculosis. Abdominal contrast-enhanced CT and MRI confirmed a large mass in the left lateral segment of the liver with heterogeneous enhancement and intrahepatic bile duct dilation (Fig. 2). The initial diagnosis of iCCA was later proven incorrect by postoperative pathology, confirming UESL. Despite the patient's age and comorbidities, radical surgery was pursued due to tumor progression after chemotherapy and multidisciplinary consensus that the benefits outweighed risks, with informed consent obtained. The patient was diagnosed with intrahepatic cholangiocarcinoma (T1bN0M0, IB stage) and underwent left hemihepatectomy, lymph node dissection, and cholecystectomy. Intraoperatively, the tumor was completely excised with minimal blood loss (300 mL).

**Fig. 3:**
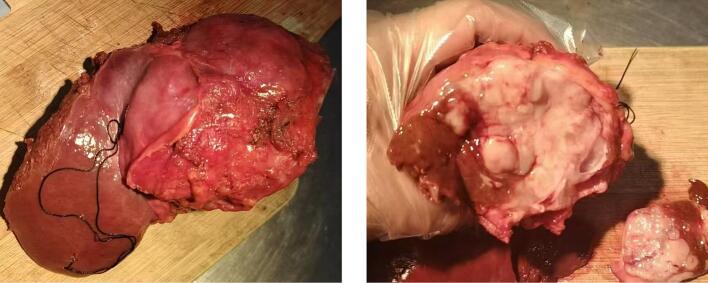
Gross specimen (A, B) revealing a solid, gray-white tumor with a smooth surface.

**Fig. 4:**
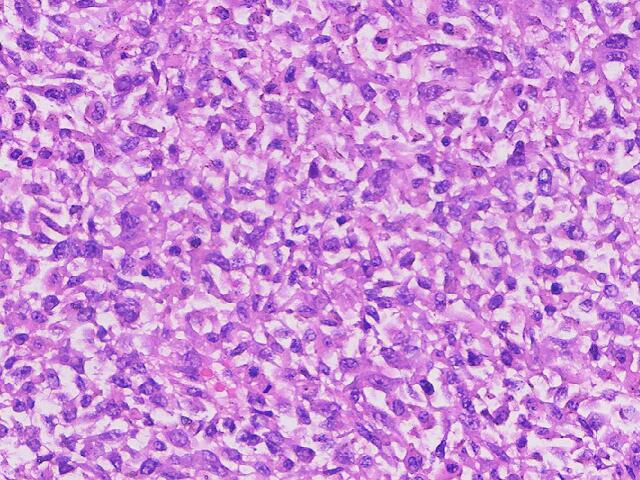
Hematoxylin and Eosin (H&E) stain demonstrating pleomorphic tumor cells with eosinophilic globules and necrosis.

**Fig. 5:**
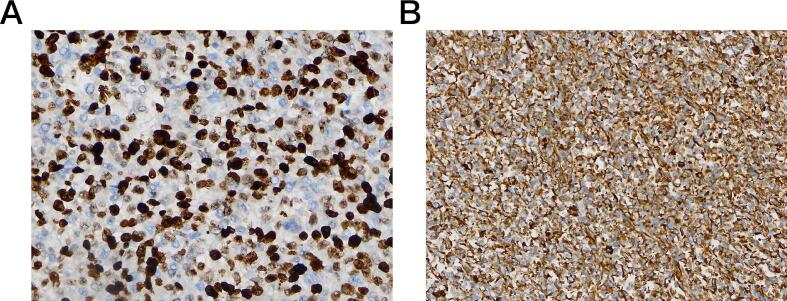
Immunohistochemistry positive staining for Ki-67 (A) and Vimentin (B).

Gross examination revealed a gray-white tumor measuring 7.5 cm × 5.5 cm × 5.0 cm, with a smooth surface and firm consistency on sectioning (Fig. 3**)**. Microscopic examination showed a sarcomatous tumor with spindle, oval, and stellate cells, high nuclear-to-cytoplasmic ratio, and frequent mitotic figures (Fig. 4). The stroma was myxoid with scattered eosinophilic globules. Immunohistochemistry was positive for Vimentin, CD10 (focal), D2–40 (focal), CD31 (focal), CD56, and β-catenin (cytoplasmic), with a Ki-67 proliferation index of 80 % (Fig. 5). The tumor was negative for epithelial markers (Pan CK, EMA, CK7, CK20, CK19, CK8, CEA), hepatocellular markers (AFP, HepPar-1), and other markers (CD34, Fli-1, ERG, CD99, S-100, Sox10, HMB45). The resection margins were free of tumor, and no lymph node metastasis was found (0/8).

The patient developed a pulmonary infection postoperatively, which was successfully treated with antibiotics. However, 1.5 months later, he presented with liver recurrence and multiple pulmonary metastases and succumbed to the disease.

## 3 Discussion

Adult UESL is a rare malignant tumor originating from primitive mesenchymal tissue in the liver, accounting for only about 7 % of all hepatic sarcomas, with a predilection for women aged 40–50 years [4]. Only ~89 adult cases were reported globally by 2021 [13], with the oldest prior patient being 86 years [11]. Our 88-year-old patient represents the oldest documented case.

Adult UESL is often asymptomatic initially; symptoms like abdominal pain/discomfort or a mass manifest with tumor growth [5]. Our patient developed vague upper abdominal pain and anorexia as the tumor enlarged. Pre-admission low-grade fever correlated with intra-tumoral hemorrhage/necrosis on CT. UESL lacks specific serological markers, though mild CA125/CA19–9 elevations occasionally occur [14,15], Here, CA19–9 rose with tumor progression.

Imaging findings are nonspecific. Ultrasound typically shows a solid mass with cystic changes [6]. CT/MRI reveals a cystic-solid lesion (often right-lobe) with necrosis, septations, and variable enhancement [3,5,16]. PET-CT demonstrated intense 18F-FDG avidity in this case, suggesting malignancy and possible nodal involvement—later disproven histopathologically (0/8 nodes), highlighting PET-CT's limitations in nodal staging for sarcomas [17].

Preoperative diagnosis is challenging due to nonspecific features, frequently leading to misdiagnosis as abscess, echinococcosis, or carcinoma [7,8]. Here, imaging and CA19–9 elevation favored iCCA over UESL. Notably, while UESL often appears cystic-solid due to necrosis [18]. This case presented as a solid mass, possibly not yet progressed to the cystic stage, lacking the CT appearance of a predominantly cystic solid-cystic mass commonly reported for UESL. This was a key reason for the misdiagnosis in this case.

To address such diagnostic dilemmas, we recommend early percutaneous liver biopsy for atypical liver masses, especially when imaging suggests iCCA but lacks confirmatory features, as supported by literature [3,6,7]. Multidisciplinary consultation with hepatobiliary specialists and consideration of advanced techniques like contrast-enhanced ultrasound or molecular profiling could aid differentiation [8,9]. In our case, a pre-chemotherapy biopsy might have confirmed UESL earlier, potentially altering the treatment plan to upfront surgery and avoiding chemotherapy delay. However, biopsy was not pursued due to perceived risks in this frail elderly patient. Limitations include reliance on imaging alone and the patient's comorbidities (e.g., pulmonary tuberculosis history), which contributed to rapid metastasis and death despite resection.

Adult UESL is highly malignant, grows rapidly, and is prone to postoperative recurrence and metastasis. The clinical prognosis is worse than in pediatric patients. The 5-year overall survival rate for pediatric UESL can reach 84.4 %, compared to only 48.2 % for adult UESL [13]. Extrahepatic metastasis occurs in 5 %–15 % of patients, commonly involving the lungs, diaphragm, and peritoneum, with adult patients being more susceptible to distant metastasis than children [9]. Elderly patients face additional challenges, including reduced tolerance to adjuvant therapy and higher frailty, exacerbating poor outcomes. This patient developed intrahepatic recurrence and bilateral lung metastases within one-month post-surgery, leading to death with an overall survival time of about one year, consistent with early literature reports [1]. Currently, radical surgical resection is the primary treatment for adult hepatic UESL. Achieving a negative resection margin is a crucial prognostic factor. Postoperative adjuvant chemotherapy significantly improves overall survival and disease-free survival rates [4,9]. Liver transplantation offers a potential treatment option for patients with localized UESL deemed unresectable [10]. For future cases, we suggest routine immunohistochemical profiling for sarcomas in atypical resections, post-operative surveillance with CT/MRI every 3 months, and individualized adjuvant regimens considering patient age and comorbidities.

## 4 Conclusion

UESL in the elderly is exceedingly rare. Its clinical presentation and serological findings lack specificity, making preoperative diagnosis challenging and prone to misdiagnosis. Pathological examination remains essential for definitive diagnosis. The prognosis for elderly UESL patients is poor. Evidence from literature supports early radical resection as the best curative option when feasible, with adjuvant therapy improving outcomes in select cases [4,9,10]. However, in elderly patients, treatment must be individualized, balancing surgical risks against aggressive tumor biology and comorbidities. Further research and improved understanding are crucial for enhancing the recognition of UESL in the elderly. Early diagnosis and prompt radical resection surgery are key to achieving a favorable prognosis for elderly patients.

## Author contribution

**Xiang-Hui Geng:** Conceived and designed the study, led the research team and contributed to manuscript revision.

**Deng-Shuai Li:** Assisted in data collection and analysis.

**Xing-Fu Wang:** Assisted in data collection and analysis.

**Wei Zhong:** Assisted in data collection and analysis.

**Min-Feng Liang:** Provided critical revisions that were important for the intellectual content.

**Jie Lin:** Coordinated the research efforts, ensured the integrity of the work, and contributed to manuscript preparation and submission.

## Informed consent

Written informed consent was obtained from the patient's son for publication of this case report and accompanying images. A copy of the written consent is available for review by the Editor-in-Chief of this journal on request.

## Ethical approval

This study was approved by Ethics Committee of The People's Hospital of Jiashi County (JSYY-HEC-202542).

## Research registration number

Not applicable.

## Declaration of Generative AI and AI-assisted technologies in the writing process

AI-assisted tools were not used in this research.

## Funding

None declared.

## Declaration of competing interest

The authors declare no conflicts of interest.
